# Influence of masticatory function on food preferences and cognitive performance in centenarians: an observational study

**DOI:** 10.1038/s41405-025-00321-z

**Published:** 2025-03-28

**Authors:** Katia Rupel, Matteo Biasotto, Filomena Vella, Giulia Ottaviani, Roberto Di Lenarda, Mauro Tettamanti, Gabriella Marcon

**Affiliations:** 1 Department of Medicine, Surgery and Health Sciences, University of Trieste, Trieste, Italy; 2 Azienda Sanitaria Universitaria Giuliano Isontina (ASUGI), Trieste, Italy; 3 Azienda Sanitaria Friuli Occidentale (ASFO), Pordenone, Italy; 4 Istituto di Ricerche Farmacologiche Mario Negri IRCCS, Milano, Italy; 5 Department of Medicine, University of Udine, Udine, Italy

**Keywords:** Gerodontics, Occlusion

## Abstract

**Introduction:**

Demographic projections forecast that centenarians’ population growth will continue. “CaT: Centenari a Trieste” is an ongoing study featuring the collection of demographic and anamnestic data, including the analysis of oral variables.

**Aims:**

evaluate of the possible influence of past food preferences, taste perception and masticatory function on current cognitive status in a cohort of centenarians.

**Design:**

Observational transversal study.

**Materials and Methods:**

31 participants with mean age 102 ± 2 performed an examination of the oral cavity including the measurement of DMFT (Decayed Missing Filled Teeth), FTUs (functional teeth units), FOUs (functional occlusal units) and 6-n-propylthiouracil (PROP) taste perception assay. Results were correlated with dementia, subjective perception of oral health and food preferences.

**Results:**

Masticatory function did not correlate with dementia but had a significant impact on oral health perception. When analyzing variables affecting food preferences, PROP taste perception profile and DMFT resulted significantly correlated. Early edentulism didn’t show to affect past dietary preferences.

**Conclusions:**

our data suggest that in centenarians masticatory function doesn’t seem to correlate to cognitive function, but influences the self-perception of oral health. Such results are an interesting addition to knowledge on the topic as they refer to a population which has never been considered before.

## Introduction

Centenarians, aged 100 years or above, are one of the fastest growing age segment populations in the last decades and this will continue to grow. At the end of last century, centenarian’s prevalence in Europe was 1 per 10,000 people, nowadays their prevalence rate is around 1 per 5000 [1] and an increasingly higher percentage of individuals born after the year 2000 will probably live more than 100 years [2]. In Italy, the number of centenarians is projected to grow from about 19.000 in 2024 to close to 80.000 subjects in 2060 [3].

A high number of population-based studies in older people demonstrate an exponential increase in age-related disorders. Among them, dementia is one of the major causes of loss of self-sufficiency in older people and it is one of the worst disabilities in terms of both economic costs of social and health spending [4, 5].

A large number of studies show that dementia is a multifactorial process in which genetics, environment and lifestyle can interact across the lifespan as either risk factors or protective factors in the development of cognitive impairment in old age. Many risk factors for dementia are modifiable [6], especially nutrition [7] that can contribute to the likelihood of healthy aging [8] and prevent low inflammation, one of the main cause of age-related disorders.

Numerous studies have shown that food preferences are one of the primary factors influencing food consumption. These preferences are deeply rooted in individual taste perceptions, which include the basic taste qualities: sweet, sour, bitter, salty, and umami. The liking for these tastes shapes individuals’ food choices and eating habits, guiding them toward certain foods [9, 10]. but little is known about the correlation between interindividual variation in the perception of taste and the intake of specific food patterns [11–13]. Most of the knowledge in the field involves the genetic and phenotypic profile of bitter taste perception, mediated by a family of TAS2R taste receptors. Specific polymorphisms in the TAS2R38 gene have been associated with increased bitter taste perception of phenylthiocarbamide (PTC) and 6-n-propylthiouracil (PROP) and is believed that it impacts on food preferences and eating behavior influencing nutritional status and general health [14–16]. Few studies have described the impact of aging on PROP sensitivity: while a study found a decrease in the rate of supertasters above the age of 70 [17], in our previously published study we found a higher proportion of supertasters among a cohort of centenarians [18].

Food preferences can be influenced also by the masticatory ability of older subjects, and oral health in general. In fact, the older population is more likely to have less natural teeth, and often wear defective dentures which lead to a worse masticatory performance possibly influencing chewing and nutrition [19], social activities [20] and cognitive status [21]. The impact of missing teeth or inadequate occlusal rehabilitation on chewing ability is being closely studied. Reduced mastication lowers the release of specific mediators from masticatory muscles that support neuronal activity and amyloid plaque clearance in the hippocampus. It also activates neurons in the Locus Coeruleus, which play a role in neuroinflammation and neurotrophic support. Mastication increases blood flow to areas often affected by cognitive decline, suggesting that stimulating these areas could help prevent deterioration. However, linking chewing function directly to cognitive health is challenging due to the complexity of the association and long-term effects. Indirect factors, like mastication’s role in digestion, stress reduction, and social interactions, are also important for maintaining cognitive function [21]. Considering the fact that previous studies did not consider specifically participants older than 100 years, the possible association between oral health, masticatory function and cognitive status in centenarians is still to be disclosed.

“Centenarians in Trieste Study” is an ongoing study featuring the collection of demographic and anamnestic data, neurological and neuropsychological examinations, which recently have included also the analysis of oral cavity and mouth-related variables.

Here we report the evaluation of the possible relation between food preferences, taste perception and masticatory function and cognitive status in a cohort of centenarians.

## Methods

### Participants and ethical considerations

Thirty-one centenarians enrolled in the “Centenarians in Trieste Study” [22] gave consent to participate in the evaluation of the oral cavity in addition to the neurological, neuropsychological and cardiological examination. The study was approved by the Friuli-Venezia Giulia Regional Ethics Committee (CEUR-2016-Em-097-ASUITS). Written informed consent was signed directly by the study participant, or by his or her legal guardian or support administrator in case of cognitive impairment, in accordance with the principles outlined in the Declaration of Helsinki. The following variables were recorded interviewing the participants and/or their caregivers and searching their records: age, gender, systemic diseases.

### Dietary preferences

A questionnaire was prepared regarding past dietary habits and preferences. Specifically, participants were asked to answer whether they used to eat specific foods (cooked vegetables, raw vegetables, meat, cured meats, pasta, rice, sweets, fruits, cheese, milk and fish). Considering the possible impairment in the accuracy of the responses in participants with cognitive decline, they were assisted by family members/caregivers when needed to validate the answers, and only those whose cognitive capacities were sufficient to undertake the questionnaire were included in the analysis.

### Cognitive variables

The diagnosis of dementia was documented combining neurological examinations and cognitive assessment. Particularly, neuropsychological evaluation for cognitive impairment was performed using Mini-Mental State Examination, Clinical Dementia Rating Scale, and, whenever possible, performing a series of seven tests taken from the Consortium to Establish a Registry on Alzheimer’s Dementia battery [23]. The diagnosis of dementia was made applying the criteria of the Diagnostic and Statistical Manual of Mental Disorders-4th edition [24].

### Oral variables

A comprehensive examination of the oral cavity was conducted by a dentist, specifically trained in the oral medicine and pathology field. The examination included inspection and palpation procedures performed using adequate external lighting, a mouth mirror and sterile gauzes. The number of residual teeth and the presence of fixed or removable prosthesis were recorded. The number of functional residual teeth was determined excluding teeth with severe caries/periodontal disease or partially erupted so they were not used for mastication. In case of edentulism, participants were asked to report the number of years in this condition. Those with at least 30 years of edentulism were categorized as “Early edentulism” in order to evaluate a possible correlation with past food preferences. The Decayed Missing Filled Teeth (DMFT) index, which is the sum of the number of decayed, missing and filled among the permanent teeth and is considered among the most important indexes for assessing the status of oral and dental health, was calculated as previously described [25].

The masticatory function was evaluated using Functional Tooth Units (FTUs), either with or without considering removable prosthesis, and Functional Occlusion Units (FOUs) as further explained. FTUs are defined as pairs opposing natural or artificial teeth, were calculating 2 points for each pair of intercuspated molars and 1 point for each pair of premolars [26], with a maximum number of 12. Considering that in our cohort a large part of participants were denture wearers, we defined a customized parameter, rpFTUs (removable prosthesis FTUs), where we included also teeth belonging to removable prosthesis (both partial and total). Therefore, rpFTUs were calculated with the same method as conventional FTUs, including both fixed teeth (both natural and artificial) and removable prostheses. FOUs were defined as a pair of opposing natural or restored teeth, including removable dentures, in contact at maximum intercuspation. This included anterior and posterior teeth which had contact with opposing teeth, with a maximum number of 14. Participants with FTUs ≥ 6 and FOUs ≥ 10 were considered as having a good masticatory performance.

Participants were asked to report self-perceived oral health both in present time and in the past (30 years before), and if they experienced discomfort in chewing and in social interactions because of their oral status.

### PROP taste evaluation

Participants were screened for PROP taste sensitivity using a filter paper disk method previously described to classify individuals by PROP taster status in general population [27]. Two filter paper disks were employed, one impregnated with NaCl (1.0 mol/L) and the other with PROP solution (50 mmol/L). Participants were asked to place the paper disk on the tip of the tongue and rate the intensity of the taste using the labelled magnitude scale. Participants were classified as non-tasters, medium tasters or supertasters using the following cut-off scores: PROP > 67 were classified as supertasters; PROP <15 non-tasters; all other were classified as medium tasters. NaCl intensity rating was employed to assign participants to each category in case of borderline ratings. Only the participants whose cognitive capacities were considered sufficient to undertake the evaluation were included.

### Salivary flow

Unstimulated salivary flow rate was calculated, and saliva samples were collected. Participants with unstimulated salivary flow <0.1 ml/min were diagnosed with “Objective xerostomia” [28], whereas those complaining with symptoms of dry mouth were assigned to the “Subjective xerostomia” group. Participants were asked to fast for at least 2 h before sampling, which was performed mid-morning 3 h after breakfast in all participants with a standard protocol. Unstimulated whole saliva was collected into pre-weighted Falcon tubes (BD Falcon^TM^ conical tubes, BD Biosciences, Two Oak Park, Bedford, MA 01730 USA) via the spitting method for 5 min while the participant was sitting in an upright position with the head slightly tilted forward and the eyes open. The tubes were subsequently re-weighted, and the salivary flow was measured applying the formula: (tube with sample – empty tube)/1.007/5 and expressed as ml/min.

### Statistical analysis

The Prism 6.0 software (GraphPad Software, La Jolla, California, USA) and R software, version 4.0.2 (R Foundation for Statistical Computing, Vienna, Austria. http://www.R-project.org/) were used to perform statistical analysis. All statistical assessments were two-sided, and a *p*-value < 0.05 was used for the rejection of the null hypothesis. Characteristics of the participants were described using means and standard deviations (SD) for numerical variables, and numbers and percentages for categorical variables. Mann-Whitney’s U test and Kruskal-Wallis tests were used to assess significance of differences between groups for each numerical variable. The Chi-square test (with Yates correction since total number of participants was less than 40) was used to test the significance of the correlation of food preferences for classification variables.

## Results

### Participants included in the study

Among the 150 ultra-centenarians included in the “Centenarians in Trieste” Study up to now, the last 31 enrolled participants underwent oral health evaluation. Demographic characteristics of the participants are reported in Table 1. Fifteen centenarians (48%) met the criteria of diagnosis of dementia according to criteria set out in the protocol [22] and described in the methods section [briefly: concordance between neurologist and neuropsychologist].

**Table 1:** Demographic and oral variables of the participants.

Number of participants	31
Age (mean ± SD)	102 ± 2
Gender (*N*, %)	
Male	2 (6%)
Female	29 (94%)
Dementia (*N*, %)	15 (48%)

*SD* standard deviation, *N* number of participants.

### Correlation of food preferences to PROP taste profile and dentition characteristics

Among the 31 centenarians assessed with the PROP taste sensitivity test, 33% were super-tasters, 38% were non-tasters and 29% were medium-tasters. Results of the questionnaire on past food preferences and their correlations with oral variables are reported in Table 2. As for preferences in the past in choosing different types of food, the centenarians had a very varied diet including for almost all of them raw and cooked vegetables, fruit, pasta and milk, while to a lesser extent meat, cured meat, rice and sweets. Analyzing the PROP taste profile in relation to past food preferences, we found a significant correlation with meat (Chi-Squared test *p* < 0.05) and cured meat (Chi-Squared test *p* = 0.01), suggesting that a lower proportion of super-tasters liked eating meat, while a higher proportion of medium-tasters preferred cured meat.

**Table 2:** Association between past use of specific foods and PROP taste profile, early edentulism, DMFT and early edentulism.

Type of food	Past users, *n* (%)	PROP taste profile (%) non/medium/super	DMFT (mean) users/non-users	Early edentulism (%) yes/no
Pasta	24 (77)	89/71/88	24.80/24.92	88/85
Rice	17 (55)	67/71/38	23.25/26.05	75/54
Meat	19 (61)	67/86/25*	25.10/24.79	75/62
Cured meat	13 (42)	11/86/43*	25.13/25.07	38/42
Fish	19 (61)	78/71/50	25.30/24.68	75/69
Cheese	20 (65)	56/86/75	24.78/24.95	63/77
Cooked vegetables	28 (90)	100/86/100	25.36/12.00*	100/92
Raw vegetables	31 (100)	100/100/100	25.10/NA	100/NA
Sweets	5 (16)	22/29/0	24.88/25.00	25/41
Milk	24 (77)	100/86/63	28.00/24.25*	88/85
Fruits	28 (90)	100/100/88	24.79/28.00	100/92

*p < 0.05.

Pearson’s chi-squared test for categorical variables and Mann-Whitney U test and Kruskal-Wallis test for numerical variables.

When analyzing if difficulties in chewing caused by early edentulism may have conditioned the dietary preferences of participants, we didn’t find significant differences in the composition of past diet between early edentulous and not edentulous participants (Chi-Squared test *p* = NS). The DMFT is a commonly used numerical index which indicates the development of dental caries, thus reflecting the deterioration of oral hygiene over time. Statistical analysis showed how participants with higher mean DMFT (indicating major loss of teeth) were significantly correlated with cooked vegetables and milk preference (Mann-Whitney U test *p* < 0.05).

When evaluating possible variations of the diagnosis of dementia in relation to PROP taste profile or food preferences, we didn’t observe significant differences (Kruskal-Wallis test *p* = NS and Chi-Squared test *p* = NS).

### Correlation of dentition and masticatory function to cognitive and subjective factors

The mean number of residual teeth in our population of centenarians was 5 ± 8, with 71% of completely edentulous participants, and 58% wearing removable dentures. 38% of participants were edentulous for more than 30 years. 29% of participants suffered from objective hyposalivation (with an unstimulated salivary flow rate <0.1 ml/min), while 41% reported to suffer from subjective sensation of dry mouth. Of notice, there was not a significant correlation between subjective and objective xerostomia (Chi-Squared test p=NS).

Mean number of FTUs was 0.6 ± 1.7, with only one participant with a number of FTUs > 6 (which was the cut-off value for good chewing performance). When also removable dentures were considered for calculation, we found a mean number of rpFTUs of 6.3 ± 5.6, with 52% of participants having a number of rpFTUs > 6. Considering both anterior and posterior occluding teeth, the mean number of FOUs was 8.5 ± 6.1 with 55% of participants having a number of rpFTUs >10.

Considering subjective perception of oral health, 58% of participants answered to the questionnaire reporting a current good/excellent oral health, while 71% considered their oral health good/excellent in the past (30 years before). 48% of included centenarians were diagnosed with dementia. 52% of participants reported discomfort in chewing thus potentially conditioning their dietary choices, and 28% reported impairment in social activities due to a sense of embarrassment connected to the appearance and state of their mouth and teeth.

Table 3 describes the results of correlation analysis of dentition and masticatory function with cognitive status and subjective considerations about oral health as described above. We did not observe significant associations between dentition and masticatory performance–related variables and subjective perception of current oral health, discomfort in chewing, discomfort in social activities or dementia (Chi-Squared test *p* = NS). On the other hand, there are significant correlations between the subjective perception of oral health in the past and both FTUs (Mann-Whitney U-test *p* = 0.0449) and the number of residual teeth (Mann-Whitney U-test *p* = 0.0027). In both cases, participants with a higher number of FTUs and residual teeth were more satisfied with their oral health in the past. Subjective perception of oral health in the past was significantly correlated also to edentulism (Chi-Squared test *p* = 0.0149) and the presence of removable prosthesis (Chi-Squared test *p* = 0.0149). In both cases, none of the participants without edentulism and removable prosthesis reported to be scarcely satisfied with their mouth.

**Table 3:** Association between dentition and masticatory function with cognitive status and subjective perception of oral health.

Oral variable	FTU (N)	FTU > 6 (yes, *N* = 1)	FOU (N)	FOU > 10 (yes, *N* = 17)	rpFTU (N)	rpFTU > 6 (yes, *N* = 16)	Residual teeth (*N*)	Edentulia (yes, *N* = 20)	Removable prosthesis (yes, *N* = 18)
Subjective good oral health – current (mean, %)									
Yes	0.56	100%	8.00	53%	5.61	56%	6.39	50%	61%
No	0.69	0%	9.07	47%	7.15	44%	3.08	50%	39%
*p*-value	NS	NS	NS	NS	NS	NS	NS	NS	NS
Subjective good oral health – past (mean, %)									
Yes	1.12	100%	9.41	62%	6.47	60%	8.82	53%	53%
No	0.00	0%	12.43	38%	10.29	40%	0.57	47%	47%
*p*-value	**0.0449**	NS	NS	NS	NS	NS	**0.0027**	**0.0149**	**0.0149**
Discomfort in chewing (mean, %)									
Yes	0.27	0%	8.47	53%	6.67	50%	2.53	60%	56%
No	1.07	100%	9.64	47%	6.71	50%	8.36	40%	44%
*p*-value	NS	NS	NS	NS	NS	NS	NS	NS	NS
Discomfort in social interactions (mean, %)									
Yes	1.13	0%	7.75	24%	5.63	19%	5.63	33%	22%
No	0.48	100%	9.52	76%	7.10	81%	5.24	67%	78%
*p*-value	NS	NS	NS	NS	NS	NS	NS	NS	NS
Objective xerostomia (mean, %)									
Yes	0.00	0%	4.78	18%	4.00	19%	0.67	36%	28%
No	0.86	100%	9.95	82%	7.18	81%	6.77	64%	72%
*p*-value	**0.0460**	NS	NS	NS	NS	NS	**0.0061**	NS	NS
Subjective xerostomia (mean, %)									
Yes	0.33	0%	8.33	41%	6.58	44%	3.50	45%	56%
No	0.88	100%	9.53	58%	6.77	66%	6.65	55%	44%
*p*-value	NS	NS	NS	NS	NS	NS	NS	NS	**0.0474**
Dementia (mean, %)									
Yes	0.60	0%	9.13	47%	6.73	50%	5.13	50%	50%
No	0.63	100%	7.81	53%	5.81	50%	4.88	50%	50%
*p*-value	NS	NS	NS	NS	NS	NS	NS	NS	NS

*FTU* Functional Tooth Units, *FOU* Functional Occlusal Units, *rpFTU* removable prosthesis Functional Tooth Units, *N* number of subjects, *NS* not significant, significant correlations are highlighted in bold.

Pearson’s chi-squared test for categorical variables and Mann-Whitney U and Kruskal-Wallis tests for numerical variables.

Objective xerostomia was also significantly related to dentition and masticatory function. Specifically, participants with higher number of FTUs (Mann-Whitney U-test *p* = 0.0460) and residual functional teeth (Mann-Whitney U-test *p* = 0.0061) were less likely to suffer from dry mouth. On the other hand, subjective sensation of dry mouth was significantly associated to the presence of removable prosthesis (Chi-Squared test *p* = 0.0474).

Variables related to salivary function (objective or self-perceived) and to masticatory performance (DMFT, number of residual teeth, FTUs, removable prosthesis, edentulism, early edentulism) were equally distributed between groups.

Furthermore, we found a significative correlation between self-perceived oral health status in the past and dementia (Chi-Squared test p = 0.02). Notably, participants reporting good/excellent oral health in the past were less likely to be diagnosed with dementia.

## Discussion

There is a continuous effort to unravel the interactions between oral health, nutrition and the aging of brain, possibly resulting in dementia.

Among the features that are likely to have an influence on progressive age-related cognitive decline, the accumulation of reactive oxygen species and inflammation are believed to be of crucial importance [29]. A recent systematic review [30] highlighted that there is evidence that adhering to Mediterranean diet (MD), characterized by consumption of cereals, fruits, vegetables and low meat consumption [31], might reduce both oxidative stress [32] and inflammation [33], thus reducing the risk for the development of chronic diseases including dementia [34].

The adherence to dietary recommendations and food preferences in general are the result of complex interactions between genetic and environmental factors [35]. Among genetic factors, only the polymorphisms in the taste 2 receptor member 38 (TAS2R38) gene are consistently associated with significant differences in bitter (PROP) taste perception in several populations [36, 37], and little is known about genetic predisposition in the different perception of other tastes (sweet, sour, salty and umami). In the present study, we confirmed a significant influence of the PROP taste profile on food preferences in our population of centenarians, finding a lower proportion of super-tasters that liked eating meat, while a higher proportion of medium-tasters preferred cured meat. Almost all participants included raw and cooked vegetables, cereal-based foods such as pasta and rice, fruits and milk. Our results are opposite to what has been described in literature, where a non-taster profile has been associated to a lower perception of oral sensations and a lower liking of fat/sweet foods in younger populations [38, 39]. On the other hand, plant-based antioxidant molecules such as phenols, flavonoids, isoflavones, terpenes, and glucosinolates are bitter, making them more palatable to non-taster individuals, thus potentially increasing their intake and beneficial effects [40].

In recent studies, it has been demonstrated how taste perception acuity [11], as well as the proportion of super taster individuals [17], decreases with age (from nearly 30% to <20%). Notably, we found a high frequency of super tasters (33%), according to the phenotype evaluation that was obtained by using a standardized method, which has been applied without restrictions in population with different age ranges, including older participants. In the future, a genetic characterization of the specific polymorphisms in our population will be needed to confirm this observation.

Food preferences can be influenced also by the masticatory ability of older subjects. For instance, individuals with poor dentitions and worse masticatory capacity tend to consume soft, carbohydrate-based foods [41]. We asked participants to report whether they were edentulous at the time of past dietary food preferences and we didn’t find any significant correlation of early edentulism (>30 years) to any of the considered food category. When analyzing the possible influence of the progressive oral health deterioration over time, expressed as the DMFT index, we found a preference of participants with higher mean index (worse dental health status) for cooked vegetables and milk, but not for carbohydrates. When evaluating possible correlations of taste differences to cognitive status, we didn’t observe different distributions between participants with and without dementia or according to PROP taste profile.

There is growing interest on the possible relationship between the deterioration in oral health and reduced chewing function on nutrition and the decline in cognitive status. Recent studies demonstrated how a reduced mastication efficiency may contribute to a deterioration in cognitive function, therefore encouraging oral care, both preventive and rehabilitative, in older populations [42, 43]. However, specific reports on centenarians, which represent a subpopulation among the older person with peculiar characteristics, are still missing up to date. Notably, we didn’t find significant correlations between any of the considered oral or masticatory variable and dementia, in contrast with previous studies which did not consider centenarians as a specific subgroup among older individuals [44]. Furthermore, there were no significant associations between masticatory variables and subjective discomfort in chewing or impairment in social activities. Although, we found that participants with a higher number of FTUs and residual teeth, as well as participants without complete edentulism and removable prosthesis, were more satisfied with their oral health in the past, suggesting that the retention of natural dentition through the implementation of preventive measures has a significant impact on older individuals. The reasons for this difference may stem from the nature of the study itself and the limited sample size, or it could reflect a unique adaptation and compensatory capacity within our specific cohort of centenarians. Expanding the sample size is therefore essential to address this issue.

While this study provides valuable insights into the possible correlation between masticatory function and cognitive decline in a cohort of centenarians, there are several limitations that must be considered. First, the cross-sectional design of this study limits the possibility to determine a causal association between chewing performance and cognitive status, which could affect the possibility to establish a clear causal link with the results. Additionally, the relatively small sample size (31 participants), 94% being female and from a specific geographic area, limit the generalizability of the results to the general population of centenarians. Another limitation is the use of FTUs and FOUs as variables to assess masticatory performance instead of more specific chewing efficiency functional tests, which were not addressed due to the very limited compliance of participants and the setting of evaluations (mostly nursing houses). Finally, PROP taste evaluation might be influenced by age-related loss in taste acuity, which could have further impacted the findings. In addition, some participants were diagnosed with dementia, potentially impairing their ability to accurately report taste sensations. The reliability of the answer in these participants, although a possible limitation to this test, was controlled including only those whose cognitive capacities were sufficient to undertake the evaluation. The inclusion of genetic profiling associated to taste phenotype, and a baseline evaluation of taste intensity might be included in future studies to avoid possible biases. These limitations highlight the need for future research to address these gaps and further validate the conclusions drawn.

Except for our previously published study [18], this is the first research work evaluating possible correlations of taste perception profiles, food preferences, oral and masticatory function, and cognitive status in centenarians. The results described here support the continuation of the investigation, potentially through a longitudinal cohort study that includes additional assessments, such as neuroimaging and functional chewing tests, to clarify causal associations, considering that this study’s cross-sectional design prevents definitive conclusions about causality. Conducting such a study among the oldest old is particularly important, as they are seen as a living model of healthy aging. Additionally, their steadily growing numbers highlight the need for developing and implementing specific oral care protocols to enhance both their oral and cognitive health, ultimately improving their quality of life.

## Conclusions

The preliminary data described here suggest that the masticatory function of centenarians does not appear to correlate with cognitive function, but it does influence their self-perception of oral health. Furthermore, food preferences and past diet composition seem to be influenced by the individual’s taste perception profile. These results provide an interesting contribution to the existing knowledge, as they pertain to a population that has not been previously studied. Further investigations performed through longitudinal studies, with the inclusion of additional evaluations such as MRI or neuroimaging and a detailed nutritional analysis in relation to chewing efficiency are needed to elucidate how oral health influences cognitive aging.

## Data Availability

Anonymized data can be requested upon reasonable request to the corresponding author.
